# Burial Depth and Stolon Internode Length Independently Affect Survival of Small Clonal Fragments

**DOI:** 10.1371/journal.pone.0023942

**Published:** 2011-09-01

**Authors:** Bi-Cheng Dong, Rui-Hua Liu, Qian Zhang, Hong-Li Li, Ming-Xiang Zhang, Guang-Chun Lei, Fei-Hai Yu

**Affiliations:** College of Nature Conservation, Beijing Forestry University, Haidian District, Beijing, China; University of Canterbury, New Zealand

## Abstract

Disturbance can fragment plant clones into different sizes and unstabilize soils to different degrees, so that clonal fragments of different sizes can be buried in soils at different depths. As a short-term storage organ, solon internode may help fragmented clones of stoloniferous plants to withstand deeper burial in soils. We address (1) whether burial in soils decreases survival and growth of small clonal fragments, and (2) whether increasing internode length increases survival and growth of small fragments under burial. We conducted an experiment with the stoloniferous, invasive herb *Alternanthera philoxeroides*, in which single-node fragments with stolon internode of 0, 2, 4 and 8 cm were buried in soils at 0, 2, 4 and 8 cm depth, respectively. Increasing burial depth significantly reduced survival of the *A. philoxeroides* plants and increased root to shoot ratio and total stolon length, but did not change growth measures. Increasing internode length significantly increased survival and growth measures, but there was no interaction effect with burial depth on any traits measured. These results indicate that reserves stored in stolon internodes can contribute to the fitness of the *A. philoxeroides* plants subject to disturbance. Although burial reduced the regeneration capacity of the *A. philoxeroides* plants, the species may maintain the fitness by changing biomass allocation and stolon length once it survived the burial. Such responses may play an important role for *A. philoxeroides* in establishment and invasiveness in frequently disturbed habitats.

## Introduction

Disturbance is an important component of ecosystems, and occurs at different temporal and spacial scales [1], [2]. Disturbance such as grazing, trampling, fire, flood and landslips can disrupt plant structures and modify environmental conditions. For instance, it can fragment plant clones that are formerly composed of interconnected clonal individuals (i.e., ramets) [3]–[6]. It can also unstabilize soil substrates, thus causing clonal fragments of various sizes to be buried in soils at different depths [7], [8]. The ability of clonal fragments to survive and regrowth after burial may be important for successful establishment and maintenance of plant populations in disturbed habitats.

Burial in soils may greatly affect survival and growth of clonal fragments because it can change both biotic (e.g., pathogen activities) [9]–[11] and abiotic conditions (e.g., light, temperature and moisture) [8], [9], [12] and also create a physical barrier to retard the shoots to come out [13], [14]. The survival and growth of clonal fragments buried in deeper soils may mostly rely on utilization of reserves stored in plant organs when carbohydrates cannot be provided through photosynthesis [7], [8]. However, if reserves stored in plant organs are depleted before the new shoots come out (and thus be able to photosynthesize), the fragments will face the risk to die [4], [15], [16].

As an important short-term storage organ, stolon internodes play an important role in vegetative reproduction of stoloniferous plants after clonal fragmentation, because reserves (e.g., non-structural carbohydrates and soluble proteins) stored in stolon internodes can be remobilized and reused for the recovery after disturbance [4], [17]. Increasing stolon internode length may facilitate the survival and growth of clonal fragments because internode length may be positively correlated with the amount of reserves stored [15], [16]. In this study, we ask whether reserves stored in stolon internodes will have significant effects on emergence ability, survival and growth of stoloniferous plants when fragments are buried in soils of different depths.

In recent decades, numerous studies have dealt with effects of burial on germination of seeds [18]–[20] and growth of seedlings [21]–[23] and adult plants [24]–[26], but few have tested the effects on the regeneration capacity of clonal fragments under burial [7], [8], [27]. Even little is known about the responses of stoloniferous clonal fragments to soil burial and the role of stolon internode in the regeneration capacity of fragments after burial.

We conducted a greenhouse experiment in which single-node fragments of the stoloniferous, invasive herb *Alternanthera philoxeroides* with stolon internodes of 0, 2, 4 and 8 cm long were buried in soils to a depth of 0, 2, 4 and 8 cm, respectively. We used single-node fragments as the study system because in the field heavy disturbance can cause clones of *A. philoxeroides* to break into small pieces, including single-node fragments. Specifically, we address the following two questions: (1) Does increasing burial depth in soils decrease emergence ability, survival, growth and morphology of the *A. philoxeroides* fragments? (2) Does increasing internode length increase emergence ability, survival and growth of the *A. philoxeroides* fragments more under deeper burial?

## Results

### Emergence rate, emergence time and survival rate

**Figure 1:**
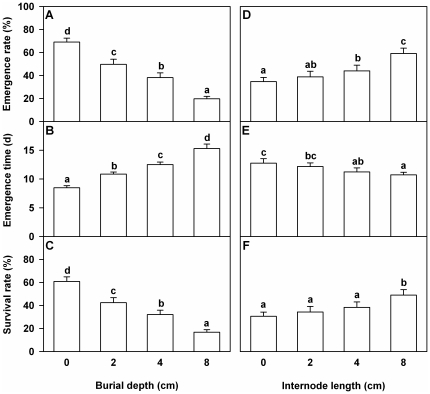
Effects of burial depth and internode length on survival of *Alternanthera philoxeroides*. Emergence rate (A and D), emergence time (B and E) and survival rate (C and F) of the *Alternanthera philoxeroides* fragments are given. Bars (A–C) in the left graphs are grand means+SE of four burial depth treatments. Bars (D–F) in the right graphs are grand means+SE of four internode length treatments. Letters show the differences between the treatments (Duncan's tests, *P* = 0.05).

Both burial depth and internode length significantly affected emergence rate, emergence time and survival rate of the *A. philoxeroides* plants (Table 1). With increasing burial depth, emergence rate and survival rate decreased markedly and emergence time increased (Fig. 1A–C). With increasing internode length, emergence rate and survival rate increased and emergence time decreased (Fig. 1D–F). However, there was no significant interaction effect of burial depth by internode length on emergence rate, emergence time or survival rate (Table 1).

**Table 1:** ANOVAs for effects of burial depth, internode length and the interaction on emergence rate, emergence time and survival rate of *Alternanthera philoxeroides*.

Effect	Emergence rate		Emergence time		Survival rate	
	DF	*F*	DF	*F*	DF	*F*
Burial (B)	3,28	29.2^***^	3,28	32.0^***^	3,28	20.9^***^
Length (L)	3,84	12.9^***^	3,79	3.9^*^	3,84	5.8^***^
B×L	9,84	1.9^ns^	9,79	0.8^ns^	9,84	1.3^ns^

Degree of freedom (DF), *F* values and the significance levels (^***^ *P*<0.001,** *P*<0.01, * *P*<0.05 and ^ns^ *P*≥0.05) are given.

### Biomass and leaf area

**Figure 2:**
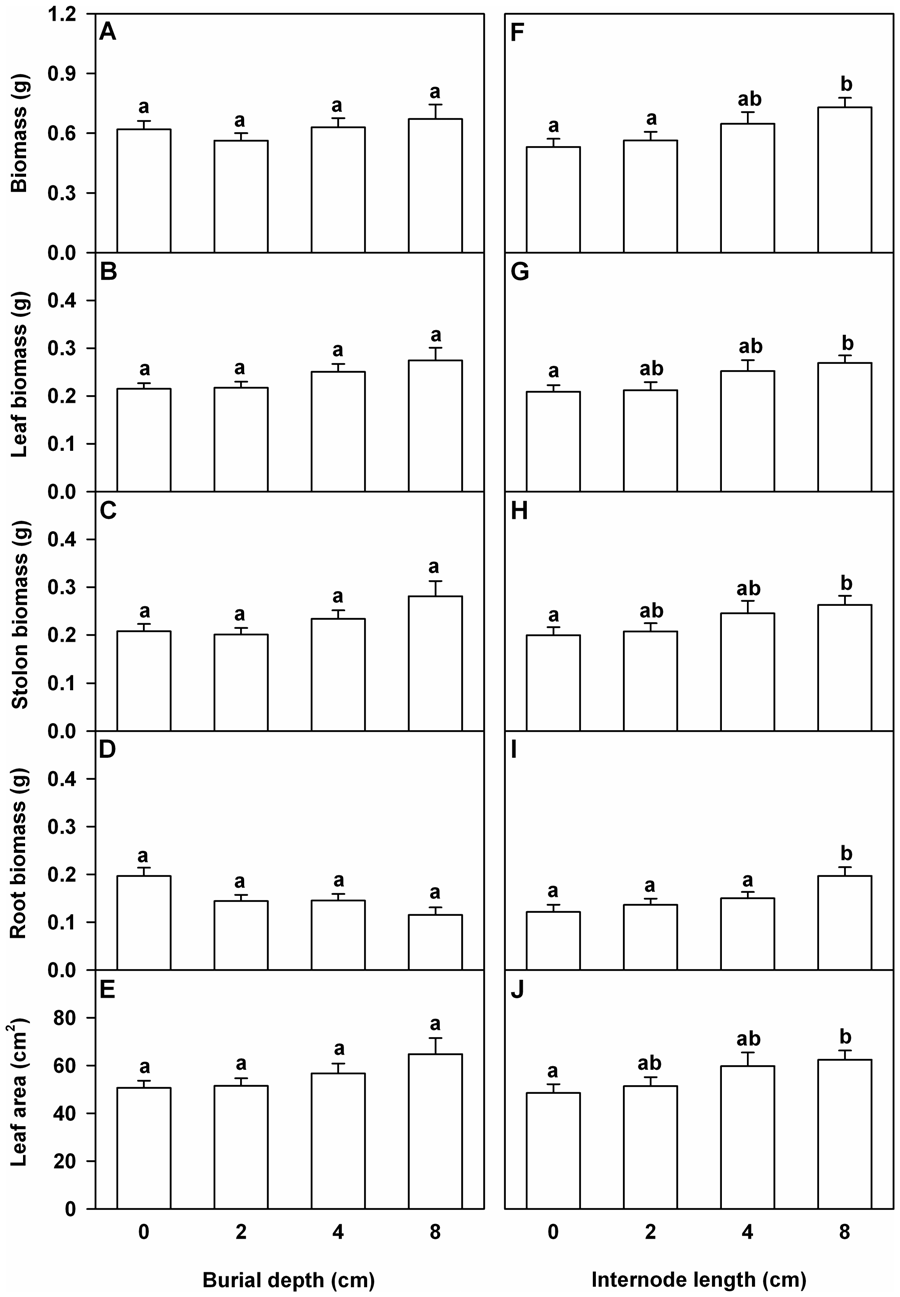
Effects of burial depth and internode length on growth of *Alternanthera philoxeroides*. Total biomass (A and F), leaf biomass (B and G), stolon biomass (C and H), root biomass (D and I) and total leaf area (E and J) of the surviving *Alternanthera philoxeroides* fragments are given. Bars (A–E) in the left graphs are grand means+SE of four burial depth treatments. Bars (F–J) in the right graphs are grand means+SE of four internode length treatments. Letters show the differences between the treatments (Duncan's tests, *P* = 0.05).

Burial depth did not significantly affect total biomass, leaf biomass, stolon biomass, root biomass or total leaf area of the *A. philoxeroides* plants (Table 2, Fig. 2A–E). Initial internode length significantly affected biomass and total leaf area of the *A. philoxeroides* plants (Table 2), and with increasing internode length total biomass, biomass of leaf, stolon and root and total leaf area increased significantly (Fig. 2F–J). There was no interaction effect of burial depth by internode length on any of the growth traits measured (Table 2).

**Table 2:** ANOVAs for effects of burial depth, internode length and the interaction on growth and morphological measures of *Alternanthera philoxeroides*.

Effect	Totalbiomass	Leafbiomass	Stolonbiomass	Rootbiomass	TotalLeafarea	Root toshootratio	Totalstolonlength	Specificstolonlength
Burial (B)	0.4^ns^	1.4^ns^	1.3^ns^	2.7^#^	1.0^ns^	17.7^***^	3.1^*^	0.8^ns^
Length (L)	6.5^***^	4.5^**^	4.7^**^	11.3^***^	4.0^**^	6.8^***^	2.3^#^	5.2^**^
B×L	1.3^ns^	1.3^ns^	1.4^ns^	1.2^ns^	0.9^ns^	1.2^ns^	1.1^ns^	0.9^ns^

*F* values and the significance levels (*** *P*<0.001, ** *P*<0.01, * *P*<0.05, ^#^ *P*<0.1 and ^ns^ *P*≥0.1) are given. Degree of freedom is (3, 28), (3, 76) and (9, 76) for effects of burial depth, internode length and the interaction, respectively.

### Root to shoot ratio, total stolon length and specific stolon length

**Figure 3:**
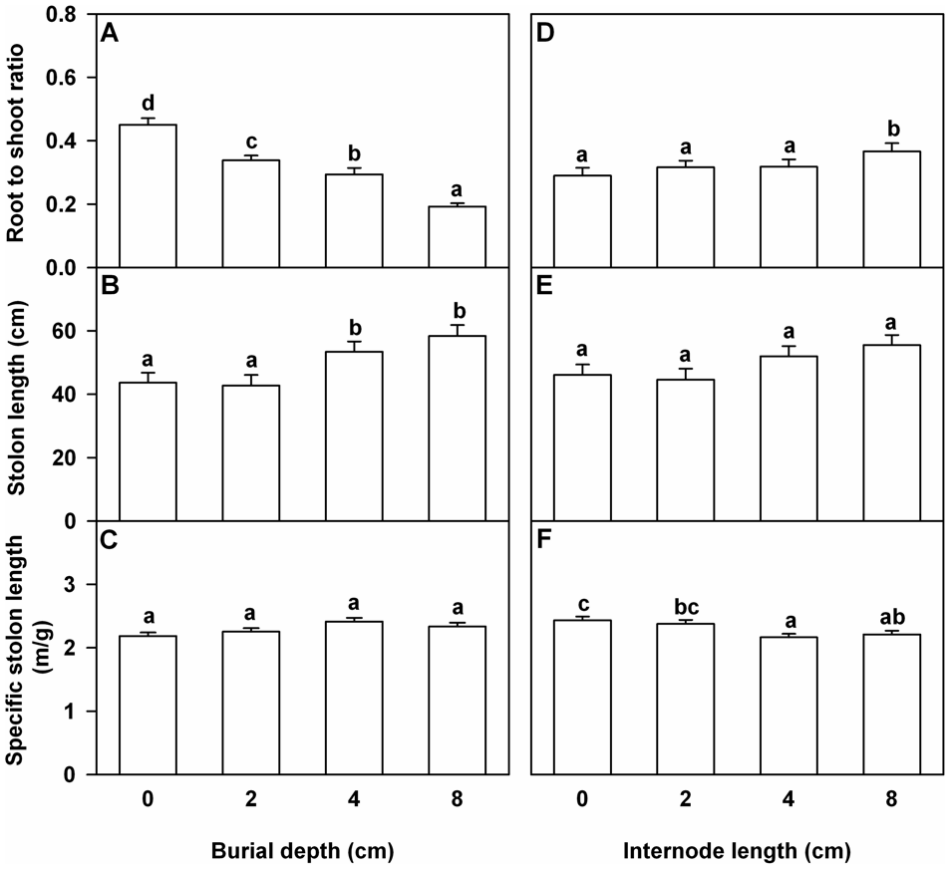
Effects of burial depth and internode length on morphology of *Alternanthera philoxeroides*. Root to shoot ratio (A and D), total stolon length (B and E) and specific internode length (C and F) of the surviving *Alternanthera philoxeroides* fragments are given. Bars (A–C) in the left graphs are grand means+SE of four burial depth treatments. Bars (D–F) in the right graphs are grand means+SE of four internode length treatments. Letters show the differences between the treatments (Duncan's tests, *P* = 0.05).

Burial depth significantly affected root to shoot ratio and total stolon length, but did not affect specific stolon length (Table 2). With increasing burial depth root to shoot ratio decreased markedly, and total stolon length increased (Fig. 3A and B). Initial internode length significantly affected root to shoot ratio and specific internode length, and marginally affected total stolon length (Table 2). With increasing internode length, root to shoot ratio and total stolon length increased or tended to increase, and specific stolon length decreased (Fig. 3D–F). There was no significant interaction effect of burial depth by internode length on root to shoot ratio, total stolon length or specific internode length of the surviving *A. philoxeroides* plants at harvest (Table 2).

## Discussion

Increasing burial depth significantly reduced the emergence ability and survival of the *A. philoxeroides* plants. Compared with the control (0-cm-deep burial), emergence time of the sprouts of the fragments in the deepest burial treatment (8-cm-deep burial) increased by 80% (15.3 days *vs.* 8.5 days) and survival rate of the fragments decreased by 44% (16.9% *vs.* 60.9%). Previous studies also found that deeper burial markedly decreased emergence and survival of rhizome fragments [7], [8], [27], [28]. One possible reason is that the physical barrier of deep burial retarded emergence of the clonal fragments, depleted the reserves stored in plant organs and thus increased mortality [13], [14].

*A. philoxeroides* could increase the biomass investment in the above-ground structure and elongate stolons to respond to burial in soils. Such responses are adaptive because they potentially increased the possibility of shoots (spouts) of the buried clonal fragments to reach above the soil surface to photosynthesize and survive, and ensured the future development of *A. philoxeroides* [12], [23], [24], [29]. Therefore, the population maintenance of *A. philoxeroides* after burial may depend closely on the optimal allocation strategy by which the buried plants increased biomass allocation to the organs that acquire the most limiting resource (i.e., light).

Burial in soils did not affect growth measures of the surviving *A. philoxeroides* plants (i.e., total biomass, biomass of leaf, stolon and root or total leaf area). However, growth responses of plants to burial are rather complex [10], [30]: burial decreases growth of some plants [29], but does not affect [10], [23], [26] or even increases growth of some others [31], [32]. There is evidence that moderate burial can improve the photosynthesis capacity and the vigor of buried plants after emergence, leading them to compensate for the consumption of storage reserves caused by burial [12], [21], [31], [33]. Therefore, one possible explanation for the irresponsiveness of the species to burial is that burial may enhance the photosynthesis rate of the emerged *A. philoxeroides* plants, and thus compensate for the loss due to the delayed emergence time or the shortened growth time (6.8 days on average) [34]. Another possible explanation is that the *A. philoxeroides* plants with low vigor (with relatively low growth rate) may have been killed by burial in the early period of plant development and the others with relatively high vigor (with high growth rate) recovered gradually from burial, resulting in that the effect of burial on growth of the surviving plants was weaken at harvest [30].

The emergence ability, survival and growth of the surviving *A. philoxeroides* plants benefited from increasing internode length. The result is consistent with previous findings [15], [16], and suggests that non-structural carbohydrates and soluble proteins stored in stolon internodes can be remobilized and reused for regeneration of plants subject to disturbance [8], [17], [35], [36]. Likewise, reserves stored in stolon internodes can contribute greatly to the fitness of the *A. philoxeroides* plants subject to burial, and thus to the successful establishment of *A. philoxeroides* in disturbed habitats. On the other hand, we also found that the *A. philoxeroides* plants originated from the longest stolon internodes fragments invested more energy in producing roots. If there are relatively stable spaces for survival in the soil layer, such biomass allocation pattern may be a strategy for rapidly recovering of *A. philoxeroides* from frequent disturbance, especially in the condition that the above-ground structure of the species is severely disrupted by disturbance [37].

We conclude that reserves stored in stolon internodes can contribute greatly to the regeneration capacity of the *A. philoxeroides* plants, whereas the regeneration capacity is severely constrained by the depths of burial in soils. At least within 8 cm deep burial the surviving *A. philoxeroides* plants might maintain the fitness by changing biomass allocation and stolon length. Such responses may play an important role for *A. philoxeroides* in establishment and invasiveness in frequently disturbed habitats.

## Materials and Methods

### The species

*Alternanthera philoxeroides* (Mart.) Griseb. is a stoloniferous perennial herb of the Amaranthaceae and originates from the Parana River region of South America [38], [39]. *A. philoxeroides* is one of the most noxious, invasive weeds in many countries, including China [40]–[42]. In the south of China *A. philoxeroides* is a common weed and widely distributed in various habitats including farmland, irrigation canals, lakesides and wastelands. In China the genetic diversity of *A. philoxeroides* is extremely low and likely originates from a single genotype [43]–[45]. *A. philoxeroides* rarely produces viable seeds and instead reproduces asexually via stolon/root fragments to sustain and renew the populations [46], [47]. Dispersal of clonal fragments can cause the rapid expansion of *A. philoxeroides* in China, resulting in great ecological and agricultural problems [44], [47].

### Material preparation and experiment design

For use in the experiment, we collected *A. philoxeroides* stolon fragments in a wasteland in the suburbs of Taizhou in Zhejiang province, China. The sampling site did not belong to the part of any farms or national parks, so we did not need any relevant permissions/permits for collecting plant samples.

On 30 July 2010, we collected over 2000 stolon fragments of *A. philoxeroides*, each consisting of an unrooted, juvenile ramet. Each ramet consisted of (i) a node with two opposite leaves, (ii) a proximal internode (before and thus developmentally older than the node) and (iii) a distal internode (after and thus developmentally younger than the node). Both proximal and distal internodes were cut into 5 cm long. On 31 July, of the 2000 stolon fragments, 1280 were selected and used for the experiment and another 60 for initial measurements. The initial stolon internode of the *A. philoxeroides* fragments was 3.73±0.05 mm in diameter (mean ± SE, *N* = 45), and the dry weight of the 0-, 2-, 4- and 8-cm-long fragments were 4.9±0.9, 12.7±0.9, 24.7±2.3, 45.3±2.4 mg (mean ± SE, *N* = 15), respectively.

The experiment took a split-plot design with burial depth as a whole plot factor and internode length as a subplot factor. There were four burial depth treatments (0, 2, 4 and 8 cm) and four internode length treatments (0, 2, 4 and 8 cm). In field stolon fragments of *A. philoxeroides* can be buried in soils deeper than 8 cm, but in this study 8 cm were used as the maximum burial depth. Each fragment consisted of both proximal and distal internodes, each having half of the total length. For instance, for the 8-cm internode length treatment, both proximal and distal internodes of the fragment were cut into 4 cm long. A total of 32 plastic containers (67.4 cm long×42.2 cm wide×17.2 cm high) were used and each was divided into four equal boxes (33.7 cm long×21.1 cm wide×17.2 cm high). Each container was filled with a 1∶1 (v∶v) mixture of sand and peat to a depth of 9 cm; total nitrogen in the soil mixture was 2.46 g kg^−1^ and total phosphorus 0.54 g kg^−1^. The four boxes within a container were randomly assigned to one of the four internode length treatments, and in each box ten fragments were placed horizontally in ten evenly spaced positions. Thus, in each container there were 40 fragments, consisting of ten fragments of each of the four internode length types (0, 2, 4 and 8 cm). Then, fragments in the 32 containers were randomly assigned to one of the four burial depth treatments, and fragments in each container were buried in soils at the same depth. The same substrate (i.e., 1∶1 sand and peat mixture) was used to cover the fragments. To keep the soil moist, enough tap water (approximate 1.5 L per container per day) was supplied.

The experiment started on 31 July 2010 and ended on 25 September. It was conducted in a heated greenhouse at Forest Science Co, Ltd of Beijing Forestry University. During the experiment the temperature and relative humidity were 24.5±0.1°C and 77.6±0.4% (mean ± SE), respectively, measured hourly by two Hygrochron temperature loggers (iButton DS1923; Maxim Integrated Products, USA).

### Measurements

During the experiment, we recorded the emergence status of each clonal fragment in each container every other day. A fragment was coded as “emerged” if the above-ground part of new shoots sprouting from the original fragment exceeded 1 cm long. On 25–28 September, we counted number of the surviving *A. philoxeroides* fragments (plants) in each box, measured total stolon length of each individual and total leaf area in each box by WinFOLIA Pro 2004a (Regent Instrument, inc., Canada). After harvest each plant was separated into leaves, stolons, roots and the original fragments, and then oven-dried at 70°C for 48 h and weighed.

### Data analyses

Because at the beginning of the experiment there were ten fragments in each box in each container, we could calculate emergence rate and survival rate of the *A. philoxeroides* fragments in each box. We also calculated the mean values of the emergence time, total stolon length, total leaf area, total biomass (the sum of leaf, stolon and root biomass), leaf biomass, stolon biomass and root biomass, root to shoot ratio (root biomass/shoot biomass; shoot mass is the sum of leaf mass and stolon mass) and specific stolon length (stolon length/stolon biomass) of the surviving plants in each box. These derived data were used for the following analyses.

The data were analyzed by split-plot ANOVA with burial depth as a whole plot factor (i.e., containers as the whole plots) and internode length as a subplot factor (i.e., boxes as the subplots) [48]. The significance of burial depth was tested against the whole plot error, whereas that of internode length and that of burial depth by internode length were tested against the subplot error. Because we detected significant main effects of burial depth and internode length but did not find a significant interaction effect on any of the traits, Duncan's tests (with *P* = 0.05) were conducted to examine for the differences among the four burial treatments and among the four internode length treatments, respectively. Because in five boxes (three in 8-, one in 4- and one in 2-cm-deep burial treatments) no fragment emerged, they were excluded for analyses of emergence time, growth and morphological measures. Also in three other boxes (one in the 2-, 4- and 8-cm-deep burial treatment, respectively) no fragment survived at harvest and they were excluded for growth and morphological measure analysis. Analyses were conducted with SPSS 16.0 (SPSS, Chicago, IL, USA).
